# HLA-DRB1*08:03-associated exocrine–endocrine immune linkage: a pathogenic mechanism in ketosis-prone type 2 diabetes

**DOI:** 10.1210/jendso/bvag200

**Published:** 2026-09-01

**Authors:** Seiya Suzuki, Yoichi Oikawa, Atsushi Satomura, Akifumi Haisa, Shumpei Nakanishi, Masashi Fujisawa, Hideo Morita, Takeshi Katsuki, Akira Shimada

**Affiliations:** Department of Endocrinology and Diabetes, Saitama Medical University, Saitama 350-0495, Japan; Department of Endocrinology and Diabetes, Saitama Medical University, Saitama 350-0495, Japan; Department of Endocrinology and Diabetes, Saitama Medical University, Saitama 350-0495, Japan; Department of Endocrinology and Diabetes, Saitama Medical University, Saitama 350-0495, Japan; Department of Endocrinology and Diabetes, Saitama Medical University, Saitama 350-0495, Japan; Department of Endocrinology and Diabetes, Saitama Medical University, Saitama 350-0495, Japan; Department of Endocrinology and Diabetes, Saitama Medical University, Saitama 350-0495, Japan; Department of Internal Medicine, Tokyo Saiseikai Central Hospital, Tokyo 108-0073, Japan; Department of Endocrinology and Diabetes, Saitama Medical University, Saitama 350-0495, Japan

**Keywords:** carbonic anhydrase II antibody, enzyme-linked immunospot assay, insulin, interferon-γ, ketosis-prone type 2 diabetes

## Abstract

**Context:**

Unprovoked ketosis-prone type 2 diabetes (KPD) is characterized by a male predominance, young age of onset, and abrupt presentation of diabetic ketosis/ketoacidosis without precipitating factors. Although many individuals regain β-cell function after insulin therapy, some progress to insulin dependence, suggesting overlap with type 1 diabetes. We previously reported the involvement of insulin-associated peptide-specific T helper 1 (Th1) responses in KPD pathogenesis. Exocrine pancreatic inflammation has been documented in type 1 diabetes, with evidence suggesting its contribution to disease development; however, its relevance to KPD remains unclear.

**Objective:**

This cross-sectional study aimed to elucidate mechanisms underlying KPD by investigating the association between endocrine and exocrine immune responses.

**Methods:**

We recruited 54 participants with KPD, assessed clinical parameters, including human leukocyte antigen (*HLA*)-*DRB1/DQB1* genotypes, carbonic anhydrase II (CA2) antibody levels, and insulin-associated peptide-specific Th1 responses, and explored their interrelationship.

**Results:**

HLA-DRB1*08:03 frequency was significantly higher in participants with KPD than in historical healthy controls. Elevated exocrine pancreatic enzyme levels were observed in 29.6% (16/54) of cases. CA2 antibody levels were increased in HLA-DRB1*08:03-positive participants, especially those with elevated exocrine pancreatic enzyme levels, and positively correlated with insulin-associated peptide-specific Th1 responses. Th1 responses were inversely associated with serum C-peptide levels in all participants, reflecting disease activity, but not in HLA-DRB1*08:03-positive participants.

**Conclusion:**

HLA-DRB1*08:03 is possibly associated with endocrine and exocrine immune responses in KPD, mediating exocrine–endocrine immune linkage. However, it is unlikely to be directly involved in β-cell impairment via insulin-specific Th1 responses, potentially explaining reversible β-cell dysfunction in early KPD.

Unprovoked ketosis-prone type 2 diabetes (KPD) is characterized by a male predominance, young age of onset, and abrupt development of diabetic ketosis/ketoacidosis (DK/DKA) in the absence of identifiable precipitating factors. It typically occurs in obese individuals who exhibit significant weight loss, lack islet-associated autoantibodies, and recover insulin secretory capacity following appropriate intensive insulin therapy, often achieving near-normoglycemia in the early phase (1-3). Historically, KPD has been reported predominantly among individuals of African and African-American ancestry and, to a lesser extent, among other minority ethnic groups (2). A Epidemiological study showed that a substantial proportion of patients presenting with DKA are African American or Hispanic (eg, 45% African American, 44% Hispanic, and 11% Caucasian) (4). In contrast, reports from East Asian populations, including Japan, remain limited. However, we previously encountered a patient with KPD who gradually progressed to an insulin-dependent state after repeated episodes of DK/DKA (5). Similar cases have been reported elsewhere (6). Based on these observations, we hypothesized that KPD shares pathophysiological features with type 1 diabetes (T1D). To test this, we previously evaluated the peripheral frequency of insulin B-chain-related peptide-specific interferon (IFN)-γ-producing peripheral blood mononuclear cells (PBMCs) in individuals with KPD. Our analysis revealed that insulin B-chain-related peptide-specific T helper 1 (Th1)-type immune responses were detectable in 36.4% of individuals with KPD, comparable to the proportion observed in individuals with acute-onset T1D (AT1D; 38.1%) (7). Additionally, Th1-type immune responses were inversely correlated with serum C-peptide levels, potentially reflecting disease activity (7).

A recent article highlighted the potential role of exocrine immune infiltration in T1D pathogenesis (8). Building on this, we hypothesized that exocrine inflammation occurs in both individuals with T1D and those with KPD and that it contributes to KPD pathogenesis via an exocrine–endocrine immune linkage. To investigate this hypothesis, we conducted a cross-sectional analysis of 54 individuals with unprovoked KPD.

## Materials and methods

### Participants and measurements

**Figure 1:**
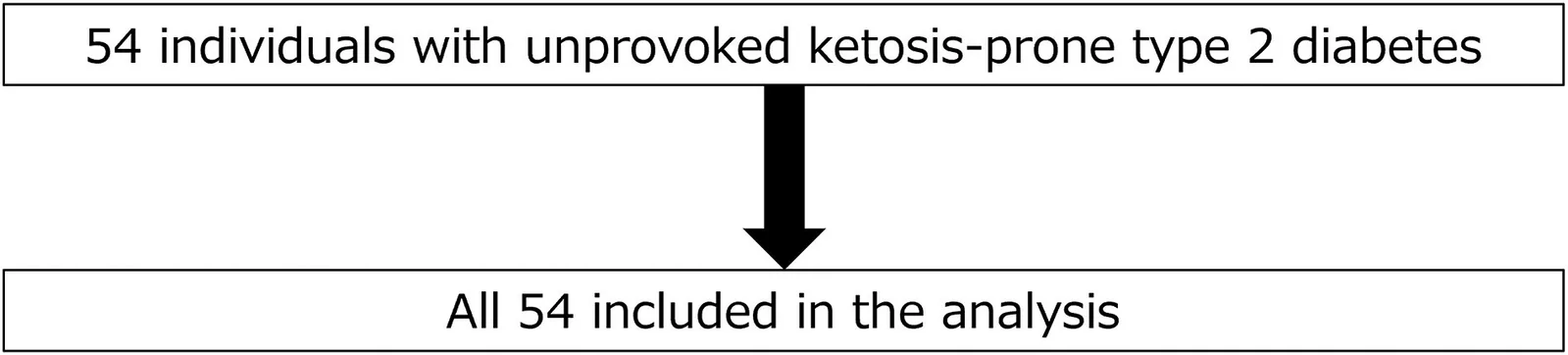
Flow diagram of participant inclusion. All 54 consecutive individuals with unprovoked ketosis-prone type 2 diabetes admitted for diabetic ketosis or ketoacidosis during the study period were enrolled. No participants were excluded.

This cross-sectional study was conducted from June 2016 to October 2025. Peripheral blood samples were obtained with written informed consent from all 54 consecutive participants (≥18 years) with KPD who were admitted to Saitama Medical University Hospital for DK or DKA during the study period (Fig. 1). Based on our previous study (7), the diagnostic criteria for KPD were defined as indicated below. (1) DK or DKA occurring within ∼3 months after the onset of hyperglycemic symptoms, such as dry mouth, polydipsia, polyuria, and weight loss. Participant interviews revealed no history of excessive soft drink consumption before symptom onset. (2) Requirement for intensive insulin therapy at diagnosis, followed by successful transition to oral hypoglycemic agents (or basal insulin) after resolution of glucose toxicity. (3) Negative results for all 4 islet-associated autoantibodies (anti-glutamic acid decarboxylase, anti-insulinoma-associated protein 2, anti-zinc transporter 8, and anti-insulin autoantibodies). (4) Relatively preserved insulin secretion capacity at DK/DKA onset, defined as fasting serum C-peptide level ≥ 0.6 ng/mL. All eligible participants were included in the analysis without exclusions.

Clinical data collected at the time of blood sampling included age, sex, body mass index (BMI), diabetes duration, ad libitum plasma glucose levels, glycated hemoglobin levels, and ad libitum serum C-peptide levels. Levels of the 4 islet-associated autoantibodies indicated above were measured as previously described (7). Additionally, serum levels of the exocrine pancreatic enzymes amylase (reference range: 37-125 U/L), lipase (reference range: 17-55 U/L), and elastase 1 (reference range: 100-400 ng/dL) were measured. The serum level of the antibody against carbonic anhydrase II (CA2), a well-established exocrine pancreatic tissue antigen in autoimmune pancreatitis (9, 10), was measured using a commercially available kit (MyBioSource Inc., San Diego, CA, USA). According to the manufacturer's instructions, both the intra-assay and interassay coefficients of variation were less than 15%. Human leukocyte antigen (*HLA*) genotyping of *HLA-DRB1* and *HLA-DQB1* alleles was performed by Wakunaga Pharmaceutical Co., Ltd. (Tokyo, Japan). Previously published data on the frequencies of HLA-DRB1*08:03 and HLA-DQB1*06:01 alleles in healthy Japanese individuals (11) were used as historical controls in this study. Additionally, historical data on the prevalence of elevated serum exocrine pancreatic enzyme levels at onset in individuals with AT1D (12) were used for comparative analysis.

### Peptide synthesis and antigens

Based on our previous reports, 4 consecutive overlapping peptides derived from the human insulin B-chain, B:9-23 (insulin B-chain peptide with amino acids 9-23; SHLVEALYLVCGERG), B:10-24 (HLVEALYLVCGERGF), B:11-25 (LVEALYLVCGERGFF), and B:12-26 (VEALYLVCGERGFFY), were chemically synthesized by Japan Bio Services Co., Ltd. (Saitama, Japan) (7, 13). The purified protein derivative of *Mycobacterium tuberculosis* (Tuberculin; National Institute for Biological Standards and Control, Hertfordshire, UK) was used as the positive control in the enzyme-linked immunospot (ELISpot) assay, as described below.

### ELISpot assay

ELISpot assay was performed using a part of ELISpot assay tools packaged in the T-SPOT.TB kit (Oxford Immunotec Limited, UK), according to the manufacturer's instructions as previously described (7, 13). Briefly, PBMCs were isolated from whole-blood samples and resuspended in the AIM-V serum-free medium supplemented with L-glutamine, gentamycin, and streptomycin (Thermo Fisher Scientific Inc., Waltham, MA, USA), adjusted to a concentration of 2.5 × 10^6^ cells/mL. In total, 2.5 × 10^5^ cells/well were seeded in 96-well plates precoated with monoclonal anti-IFN-γ capture antibody. Four insulin peptides (B:9-23, B:10-24, B:11-25, and B:12-26; hereinafter collectively referred to as “insulin B-chain amino acid 9-23-related peptides” or “B:9-23rPep”) were individually added in duplicate. Anti-human CD28 antibody (clone L293; BD Biosciences, Becton, Dickinson and Company, Franklin Lakes, NJ, USA; Cat# 348040; RRID: AB_400367) was added, and the plates were incubated at 37 °C in 5% CO_2_ for 24 hours. PBMCs cultured in the medium alone or stimulated with the purified protein derivative served as the negative and positive controls, respectively. Spot-forming cells (SFCs) were enumerated using an automated image analysis system, ELISpot reader (CTL ImmunoSpot S6 TATC Analyzer, CTL Analyzers LLC, OH, USA). Each SFC represented an antigen-specific PBMC producing IFN-γ. The number of antigen-induced SFCs was calculated as follows: ([mean number of SFCs in the presence of stimulation]/[mean number of SFCs in the absence of stimulation]). Results are expressed as SFCs per 2.5 × 10^5^ PBMCs. The peripheral frequency of B:9-23rPep-specific IFN-γ-producing PBMCs in each participant was defined as the maximum number of IFN-γ SFCs among the 4 B:9-23-related peptides and used for all subsequent analyses, as previously described (7, 13).

### Ethics

This study adhered to the Declaration of Helsinki and was approved by the Institutional Review Boards of Saitama Medical University Hospital (approval no. 15123.08; approval date: October 7, 2024) and Tokyo Saiseikai Central Hospital (approval no. 164; approval date: September 4, 2024).

### Statistical analyses

All statistical analyses were conducted using the IBM SPSS Statistics software (version 23; SPSS Inc., Chicago, IL, USA). All continuous variables are represented as the mean ± standard deviation, except for serum C-peptide and CA2Ab levels and the number of SFCs, which are expressed as the median (interquartile range). Normality of data distribution was assessed using the Shapiro–Wilk test and visual inspection of histograms. Although several variables showed *P* > .05, indicating no evidence against normality, nonparametric tests were adopted because the sample size was modest and some variables exhibited skewed distributions in specific subgroups. Group comparisons of median values and proportions were performed via Mann–Whitney *U* and χ^2^ tests, respectively. Spearman rank correlation coefficient (*r_s_*) was used to assess associations between nonparametric variables. Statistical significance was set at *P* < .05. Because unprovoked KPD predominantly affects males, the recruitment of female participants was limited. Sex was not included as a factor in the statistical analyses due to the small number of female participants.

A post hoc power analysis was performed using G*Power 3.1.9.7 (Heinrich Heine University, Düsseldorf, Germany), an openly accessible program available online, for 2-tailed correlation tests. With a sample size of 54 participants and α = 0.05, the estimated statistical power was ∼0.60 for *r_s_* = 0.30, >0.99 for *r_s_* = 0.60, and >0.99 for *r_s_* = 0.85, indicating limited power to detect small associations but sufficient power to detect strong associations.

Multivariate linear regression was performed using variables among age, sex, BMI, diabetes duration, plasma glucose, HbA1c, CA2Ab levels, and B:9-23rPep-specific ELISpot responses, selecting only those with univariate associations at *P* < .10.

## Results

### Clinical characteristics of participants with KPD

Clinical characteristics of participants with KPD (n = 54) are summarized in Table 1. The mean (± standard deviation) age and duration of diabetes were 41.9 (±13.4) and 1.1 (±3.2) years, respectively, indicating a relatively young cohort with a short disease duration. Most participants were male (51 of 54), consistent with the known male predominance in KPD. Although glycemic control was poor at the time of admission due to DK/DKA, serum C-peptide levels were relatively preserved. The frequencies of HLA-DRB1*08:03 and HLA-DQB1*06:01 were 24.1% (13/54) and 42.6% (23/54), respectively. Notably, all participants carrying HLA-DRB1*08:03 were concomitantly positive for HLA-DQB1*06:01, indicating the formation of the HLA-DRB1*08:03-DQB1*06:01 haplotype.

**Table 1:** Clinical characteristics of study participants

	Participants with KPD (n = 54)
Demographic characteristics	
Age (years)	41.9 ± 13.4
Sex (men/women)	51/3
Diabetes duration (years)	1.1 ± 3.2
BMI (kg/m^2^)	28.8 ± 4.3
Laboratory findings at the time of sampling	
Ad libitum plasma glucose level (mmol/L)	19.7 ± 7.7
(mg/dL)	354.6 ± 139.4
HbA1c (mmol/mol)	113.5 ± 13.4
(%)	12.5 ± 1.2
Ad libitum serum C-peptide level (nmol/L)	0.76 (0.48-0.98)
(ng/mL)	2.29 (1.46-2.95)
*HLA*	
Presence of HLA-DRB1*08:03 (yes/no)	13/41
Presence of HLA-DQB1*06:01 (yes/no)	23/31

Data for continuous variables, except serum C-peptide levels, are represented as the mean ± standard deviation. Serum C-peptide levels are shown as the median (interquartile range).

Abbreviations: BMI, body mass index; HbA1c, glycated hemoglobin; *HLA*, human leukocyte antigen; KPD, ketosis-prone type 2 diabetes.

### Elevated exocrine pancreatic enzyme levels and frequencies of *HLA-DRB1/DQB1* alleles in participants with KPD

Elevated serum exocrine pancreatic enzyme levels, defined as values above the upper limit of the reference range for at least 1 enzyme (amylase, lipase, or elastase 1), were observed in 29.6% (16/54) of participants with KPD, primarily due to elevated serum lipase levels, which were noted in 25.9% (14/54) of participants with KPD. This frequency was higher than the historical rate previously reported in participants with AT1D at diagnosis (11.6%; 5/43) (12); however, the difference was not statistically significant based on the χ^2^ test. Elevated serum amylase and elastase 1 levels were observed in 5.6% (3/54) and 3.7% (2/54) of participants with KPD, respectively.

In terms of *HLA* class II, HLA-DRB1*08:03 frequency was significantly higher in participants with KPD than in historical healthy controls (13/54 vs 69/794; *P* < .01 [χ^2^ test]) (11). Similarly, HLA-DQB1*06:01 frequency was significantly higher in participants with KPD than in historical healthy controls (23/54 vs 149/792; *P* < .01 [χ^2^ test]) (11). Therefore, we selected these 2 *HLA* alleles for further analysis.

### Serum CA2Ab levels are increased in HLA-DRB1*08:03-positive participants with KPD, particularly in those with elevated exocrine pancreatic enzyme levels

**Figure 2:**
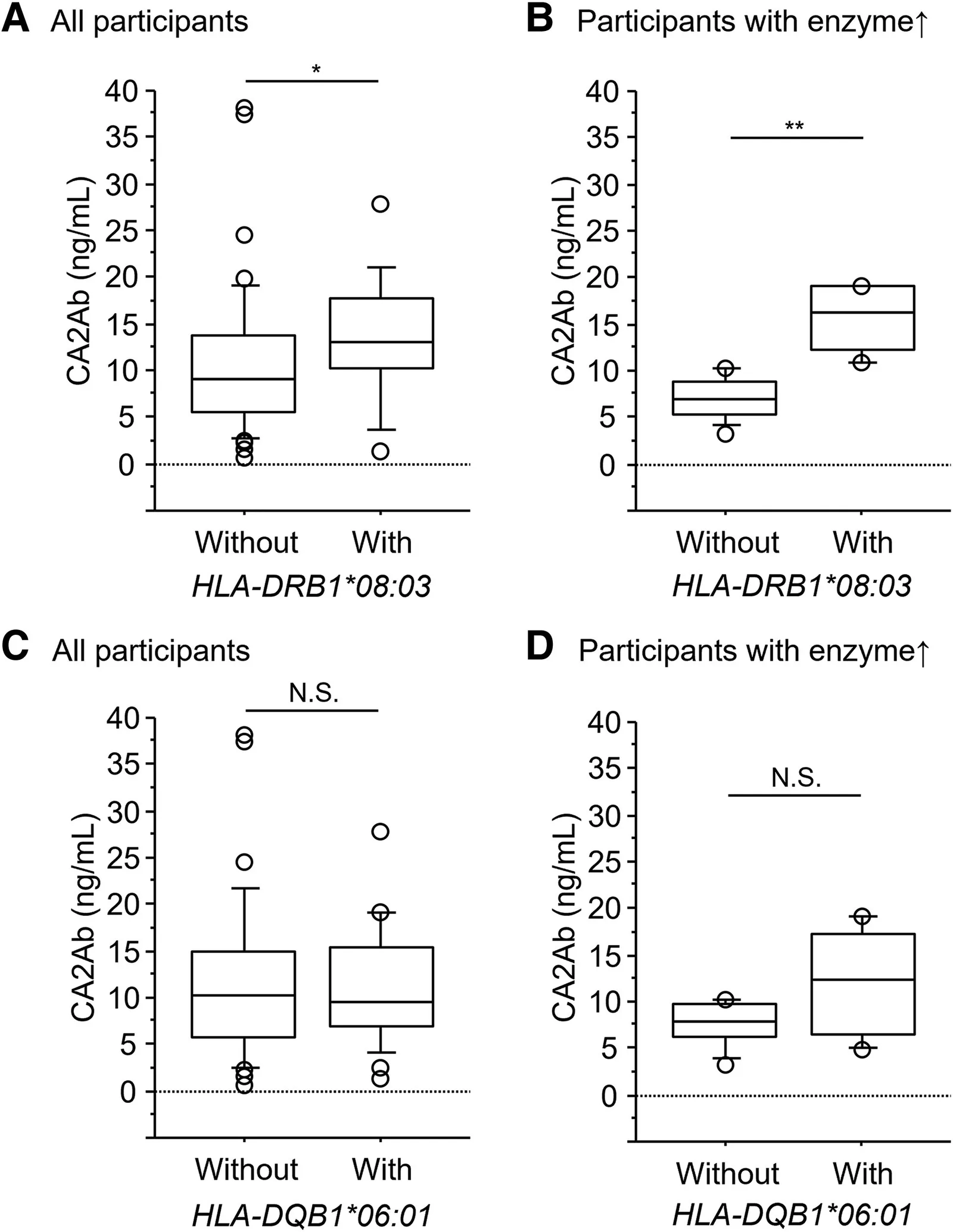
Box plots of serum CA2Ab levels in participants with KPD according to the presence or absence of HLA-DRB1*08:03 or HLA-DRB1*06:01 and the exocrine pancreatic enzyme status. All participants with KPD were divided into 2 groups: Those with HLA-DRB1*08:03 (n = 13) and those without HLA-DRB1*08:03 (n = 41). Serum CA2Ab levels tended to be higher in HLA-DRB1*08:03-positive participants than in HLA-DRB1*08:03-negative participants (A). Among participants with KPD with elevated exocrine pancreatic enzyme levels (n = 16), serum CA2Ab levels were significantly higher in HLA-DRB1*08:03-positive (n = 6) than in HLA-DRB1*08:03-negative (n = 10) individuals (B). In contrast, no significant difference in serum CA2Ab levels was observed between HLA-DRB1*06:01-positive (n = 23) and -negative (n = 31) participants (C). Similarly, among participants with KPD with elevated exocrine pancreatic enzyme levels, no significant difference in serum CA2Ab levels was observed between HLA-DRB1*06:01-positive (n = 9) and -negative (n = 7) individuals (D). The medians, interquartile ranges, upper and lower extremes, and outliers are shown. “enzyme↑” indicates elevated pancreatic exocrine enzymes, defined as values above the upper limit of the reference range for at least 1 enzyme (amylase, lipase, or elastase-1). *P < .08 and **P < .05 (Mann–Whitney *U* test). CA2Ab, carbonic anhydrase II antibody; *HLA*, human leukocyte antigen; KPD, ketosis-prone type 2 diabetes; N.S., not significant.

Next, we measured serum CA2Ab levels in participants with KPD and compared them between HLA-DRB1*08:03-positive (n = 13) and -negative (n = 41) individuals. Median (interquartile range) CA2Ab levels tended to be higher in HLA-DRB1*08:03-positive participants than in HLA-DRB1*08:03-negative participants (13.150 [9.775-18.175] vs 8.950 [5.525-13.925] ng/mL; *P* < .08 [Mann–Whitney *U* test]; Fig. 2A). When the analysis was restricted to participants with KPD with elevated exocrine pancreatic enzyme levels, CA2Ab levels were significantly higher in the HLA-DRB1*08:03-positive group (n = 6) than in the HLA-DRB1*08:03-negative group (n = 10; 16.375 [12.0125-19.1625] vs 6.875 [5.225-9.1375] ng/mL; *P* < .01 [Mann–Whitney *U* test]; Fig. 2B).

Notably, serum CA2Ab levels were comparable between participants with KPD with and without HLA-DQB1*06:01, regardless of serum exocrine pancreatic enzyme status (Fig. 2C and 2D).

### Serum CA2Ab levels are positively correlated with insulin-associated peptide-specific th1-type immune responses in participants with KPD carrying HLA-DRB1*08:03

**Figure 3:**
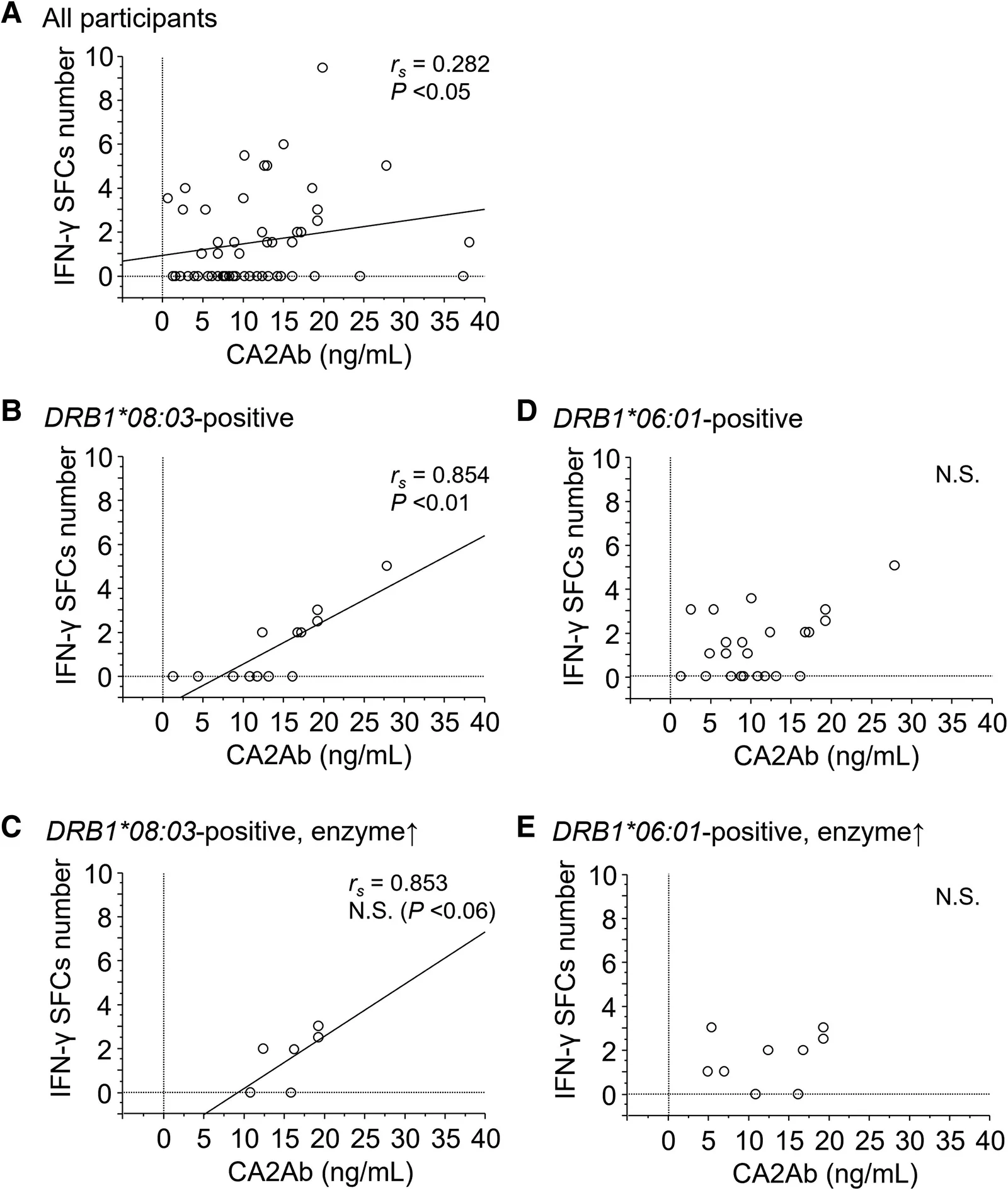
Correlations between serum CA2Ab levels and the SFC numbers of B:9-23rPep-specific IFN-γ-producing PBMCs. Dot plots show correlations between serum CA2Ab levels and the maximum number of IFN-γ SFCs in participants stimulated with each of the 4 insulin B-chain amino acid 9-23-related peptides determined via ELISpot assays. The number of IFN-γ SFCs is expressed as the number of spots per 2.5 × 10^5^ PBMCs. A significant positive correlation between the 2 parameters was observed in all participants with KPD (n = 54; Spearman correlation coefficient [*r_s_*] = 0.282; *P* < .05) (A) and those carrying HLA-DRB1*08:03 (n = 13; *r_s_* = 0.854; *P* < .01) (B). Additionally, a tendency toward a positive correlation was observed in participants with KPD carrying HLA-DRB1*08:03 with elevated exocrine pancreatic enzyme levels (n = 6) (C). In contrast, no significant correlation was observed in participants with KPD carrying HLA-DRB1*06:01 (n = 23) (D) and those carrying HLA-DRB1*06:01 with elevated exocrine pancreatic enzyme levels (n = 9) (E). “enzyme↑” indicates elevated pancreatic exocrine enzymes, defined as values above the upper limit of the reference range for at least 1 enzyme (amylase, lipase or elastase-1). B:9-23rPep, insulin B-chain amino acid 9-23-related peptides; CA2Ab, carbonic anhydrase II antibody; ELISpot, enzyme-linked immunospot; *HLA*, human leukocyte antigen; IFN, interferon; KPD, ketosis-prone type 2 diabetes; N.S., not significant; PBMC, peripheral blood mononuclear cell; *r_s_*, Spearman rank correlation coefficient; SFC, spot-forming cell.

We also investigated the association between serum CA2Ab levels and the peripheral frequency of IFN-γ-producing PBMCs specific to human insulin B-chain peptides, namely B:9-23rPep-specific IFN-γ-producing PBMCs, in participants with KPD. A weak but significant positive correlation was observed (*r_s_* = 0.282; *P* < .05; Fig. 3A). When the analysis was restricted to participants carrying HLA-DRB1*08:03, a statistically significant and strong positive correlation was observed (*r_s_* = 0.854; *P* < .01; Fig. 3B), suggesting a possible relationship between humoral and cellular immune responses in KPD. Furthermore, among HLA-DRB1*08:03-positive participants with elevated exocrine pancreatic enzyme levels, a strong trend toward a positive correlation was observed (*r_s_* = 0.853; *P* < .06; Fig. 3C). In contrast, no correlation was observed in individuals lacking the HLA-DRB1*08:03 allele (data not shown).

Notably, no correlation was observed between serum CA2Ab levels and the peripheral frequency of B:9-23rPep-specific IFN-γ-producing PBMCs in HLA-DQB1*06:01-positive participants, regardless of serum exocrine pancreatic enzymes status (Fig. 3D and 3E).

### Serum lipase levels are positively correlated with insulin-associated peptide-specific th1-type immune responses in participants with KPD carrying HLA-DRB1*08:03

**Figure 4:**
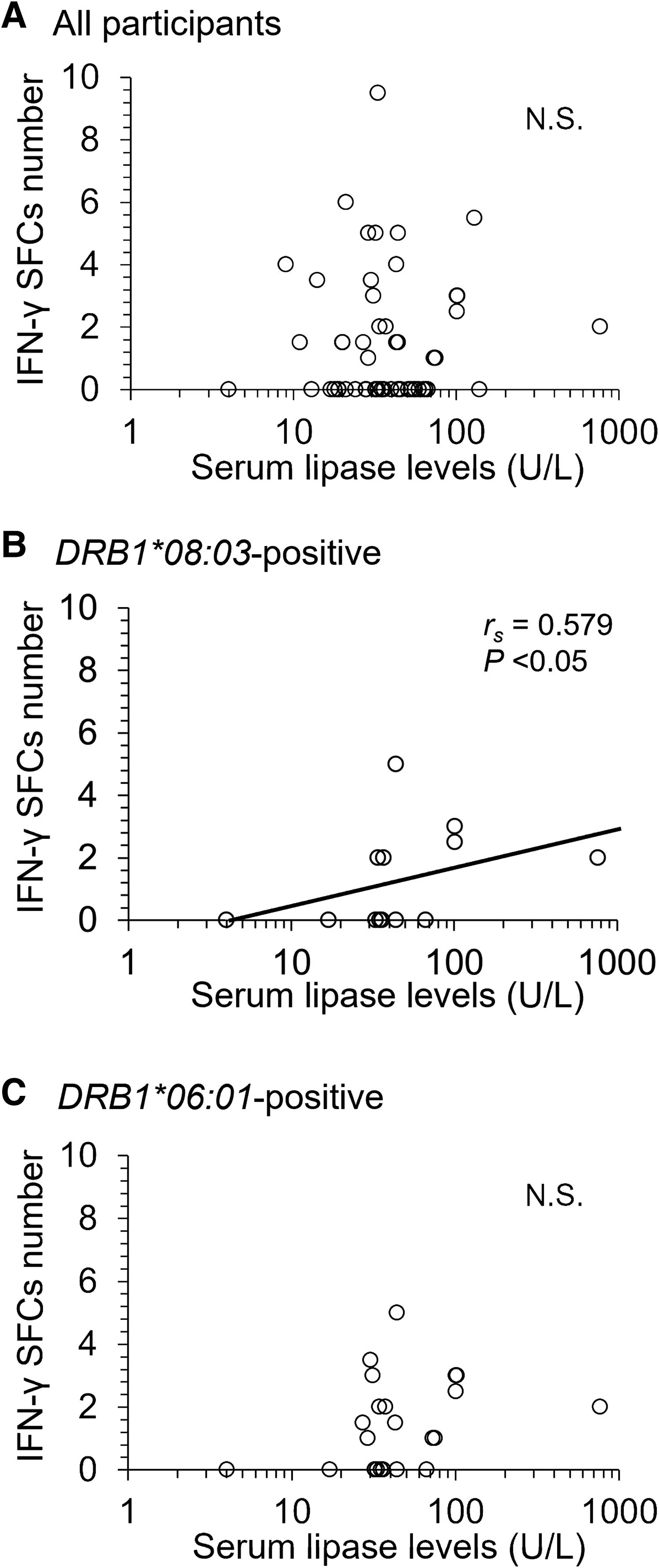
Correlations between serum lipase levels and the SFC numbers of B:9-23rPep-specific IFN-γ-producing PBMCs. (A–C) Dot plots showing correlations between serum lipase levels and the maximum number of IFN-γ SFCs in participants stimulated with each of the 4 insulin B-chain amino acid 9-23-related peptides, as determined via ELISpot assays. The number of IFN-γ SFCs is expressed as the number of spots per 2.5 × 10^5^ PBMCs. X-axis is shown on a logarithmic scale. No significant correlation between the 2 parameters was observed in all participants with KPD (n = 54) (A). In contrast, a significant positive correlation between the 2 parameters was observed in participants with KPD carrying HLA-DRB1*08:03 (n = 13; *r_s_* = 0.579; *P* < .05) (B), but not in those carrying HLA-DRB1*06:01 (n = 23) (C). B:9-23rPep, insulin B-chain amino acid 9-23-related peptides; ELISpot, enzyme-linked immunospot; *HLA*, human leukocyte antigen; IFN, interferon; KPD, ketosis-prone type 2 diabetes; N.S., not significant; PBMC, peripheral blood mononuclear cell; *r_s_*, Spearman rank correlation coefficient; SFC, spot-forming cell.

Next, we investigated the association between serum lipase levels and the peripheral frequency of B:9-23rPep-specific IFN-γ-producing PBMCs in participants with KPD. Overall, no correlation was observed (Fig. 4A). Specifically, a significant positive correlation was noted in participants carrying HLA-DRB1*08:03 (*r_s_* = 0.579; *P* < .05; Fig. 4B), whereas no correlation was observed in participants lacking this allele (data not shown). For reference, no correlation was observed between serum lipase and CA2Ab levels in the HLA-DRB1*08:03-positive subgroup (data not shown).

Moreover, no correlation was observed between serum lipase levels and the peripheral frequency of B:9-23rPep-specific IFN-γ-producing PBMCs in either HLA-DQB1*06:01-positive participants (Fig. 4C) or those without the allele (data not shown).

### Serum C-peptide levels are inversely correlated with insulin-associated peptide-specific Th1-type immune responses, but not with Serum CA2Ab levels

**Figure 5:**
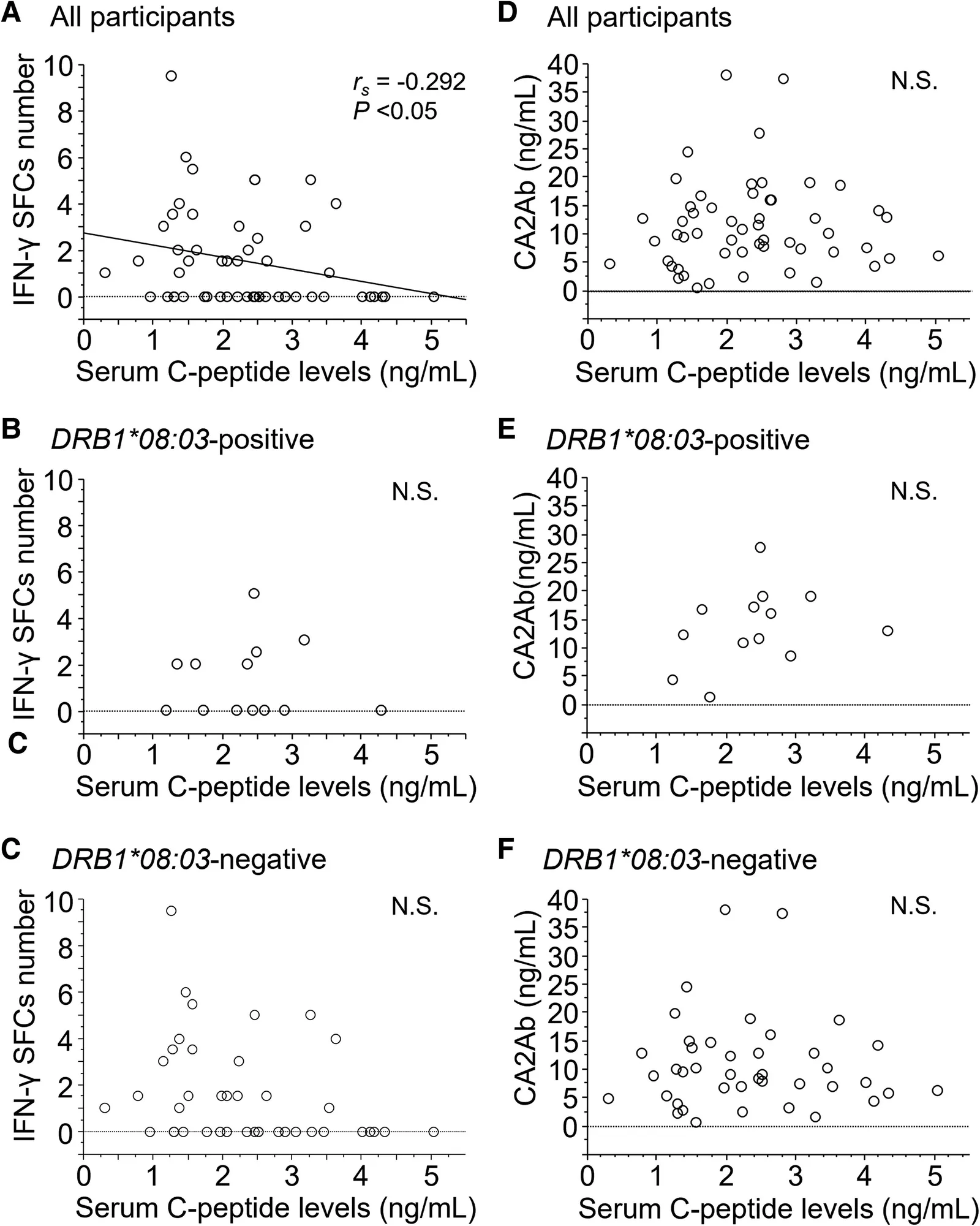
Correlations between serum C-peptide levels and the SFC numbers of B:9-23rPep-specific IFN-γ-producing PBMCs or serum CA2Ab levels. (A–C) Dot plots showing correlations between serum C-peptide levels and the maximum number of IFN-γ SFCs in participants stimulated with each of the 4 insulin B-chain amino acid 9-23-related peptides, as determined via ELISpot assays. The number of IFN-γ SFCs is expressed as the number of spots per 2.5 × 10^5^ PBMCs. A significant inverse correlation between the 2 parameters was observed in all participants with KPD (n = 54; *r_s_* = −0.292; *P* < .05) (A), but not in the HLA-DRB1*08:03-positive (n = 13) (B), and -negative (n = 41) (C) individuals. (D–F) Dot plots showing the correlation between serum C-peptide and CA2Ab levels. No significant correlation between the 2 parameters was observed in all participants with KPD (n = 54) (D), HLA-DRB1*08:03-positive individuals (n = 13) (E), and HLA-DRB1*08:03-negative individuals (n = 41) (F). B:9-23rPep, insulin B-chain amino acid 9-23-related peptides; CA2Ab, carbonic anhydrase II antibody; ELISpot, enzyme-linked immunospot; *HLA*, human leukocyte antigen; IFN, interferon; KPD, ketosis-prone type 2 diabetes; N.S., not significant; PBMC, peripheral blood mononuclear cell; *r_s_*, Spearman rank correlation coefficient; SFC, spot-forming cell.

Next, we evaluated the association between serum C-peptide levels and B:9-23rPep-specific Th1-type immune responses or serum CA2Ab levels in participants with KPD. A significant inverse correlation was observed between serum C-peptide levels and the peripheral frequency of B:9-23rPep-specific IFN-γ-producing PBMCs (*r_s_* = −0.292; *P* < .05; Fig. 5A), suggesting that B:9-23rPep-specific Th1-type immune responses contribute to β-cell impairment and reflect disease activity in KPD. However, this correlation was not evident in participants with or without the HLA-DRB1*08:03 allele (Fig. 5B and 5C). Moreover, no significant correlation was observed between serum C-peptide and CA2Ab levels (Fig. 5D), irrespective of HLA-DRB1*08:03 status (Fig. 5E and 5F), suggesting that autoimmunity against CA2 does not directly contribute to β-cell impairment in this context.

Among participants with the HLA-DRB1*06:01 allele, no significant correlation was observed between serum C-peptide levels and B:9-23rPep-specific Th1-type immune responses or serum CA2Ab levels (data not shown).

We also performed an exploratory multivariate analysis including plasma glucose and B:9-23rPep-specific Th1-type responses, the only variables that showed significant univariate associations with serum C-peptide levels (*P* < .05). No other clinical variables demonstrated meaningful univariate associations (all *P* > .10). In the multivariate model, plasma glucose remained significant (*P* < .01), whereas the ELISpot variable showed only a trend toward significance (*P* < .09). This attenuation is consistent with over adjustment, as plasma glucose directly determines serum C-peptide level. Accordingly, the multivariate model was not considered valid for further interpretation, and no additional multivariate analyses were pursued.

## Discussion

This study identified a novel association between HLA-DRB1*08:03 and potential exocrine–endocrine immune linkage in KPD. Serum CA2Ab levels appeared higher among HLA-DRB1*08:03-positive participants in the restricted subgroup with elevated pancreatic enzyme levels, which were linked to B:9-23rPep-specific Th1-type immune responses in the periphery. Additionally, a significant positive correlation was observed between serum lipase levels and B:9-23rPep-specific Th1-type responses in these individuals. These findings suggest that HLA-DRB1*08:03 contributes to the development of immune responses across both the exocrine and endocrine pancreas, thereby serving as a potential bridge between these compartments and contributing to KPD pathogenesis. To the best of our knowledge, no previous studies have examined the significance of HLA-DRB1*08:03 in KPD, underscoring the novelty of our observations.

HLA-DRB1*08:03 has been implicated in autoimmune diseases involving exocrine tissues, such as primary biliary cirrhosis (14, 15) and primary Sjögren syndrome (16), supporting its relevance to pancreatic exocrine autoimmunity targeting CA2 in KPD. CA2 is expressed in pancreatic ductal cells (9), and CA2Ab is frequently detected in individuals with T1D (17, 18). Therefore, the presence of CA2Ab suggests that autoimmunity in T1D extends beyond β-cells to involve exocrine components (17, 18). This notion is consistent with previous reports of exocrine pancreatic inflammation, predominantly involving CD8 T cells (19), impaired exocrine pancreatic function (20) and reduced pancreatic volume (21, 22) in T1D. The mechanisms of exocrine loss in T1D remain unclear; however, the leading hypotheses include developmental defects, β-cell loss resulting in exocrine atrophy, and autoimmune or inflammatory destruction of exocrine cells (23). Interestingly, CA2-positive pancreatic cells may serve as progenitors for both endocrine and exocrine compartments in animal models (24), suggesting that CA2 could link anti-islet and anti-exocrine immune responses in KPD. In line with this, we observed a positive correlation between B:9-23rPep-specific Th1-type responses and CA2Ab levels specifically in HLA-DRB1*08:03 carriers. Future studies should establish methods to detect CA2-specific T-cell responses and determine whether they correlate with insulin-peptide-specific Th1 immunity.

Consistent with our previous report (7), B:9-23rPep-specific Th1-type responses were inversely associated with insulin secretory capacity in the overall cohort, suggesting that these responses reflect disease activity. However, this inverse correlation was absent in HLA-DRB1*08:03 carriers, indicating that the exocrine–endocrine immune linkage may contribute less directly to β-cell impairment in this subgroup. The HLA-DRB1*08:03–DQB1*06:01 haplotype, which confers relative resistance to T1D in Japanese individuals (25), may underlie the reversibility of β-cell dysfunction observed after recovery from DK/DKA in early KPD. Although HLA-DQB1*06:01 was frequently detected, it showed no association with immune parameters, suggesting a role independent of insulin- or CA2-directed immunity and possibly involving other molecular targets. Overall, our findings indicate that exocrine–endocrine immune linkage is specific to HLA-DRB1*08:03 and may define a distinct immunogenetic subset of KPD.

These observations may have clinical relevance. HLA-DRB1*08:03, together with elevated CA2Ab or pancreatic enzyme levels, may serve as markers of immune activation and help identify patients with exocrine–endocrine immune linkage. Given the potential reversibility of β-cell dysfunction in early KPD, immunogenetic stratification may support individualized treatment strategies, including optimization of insulin therapy. Future studies should evaluate the utility of these immunological parameters as predictive tools in diverse populations.

This study has some limitations. First, this study was cross-sectional in design; therefore, the observed associations do not provide direct evidence of causality. Second, the sample size was relatively small, particularly in the subgroup carrying HLA-DRB1*08:03, which limited the statistical power, as demonstrated in the post hoc power analysis described in the Methods section. Therefore, our results should be interpreted with caution, and further validation in larger cohorts is warranted. Third, although 51 of the 54 participants had ad libitum serum C-peptide levels ≥1.0 ng/mL at the time of evaluation—indicating preserved endogenous insulin secretion—progression to an insulin-dependent state could not be assessed within this cross-sectional design. Fourth, T-cell responses against CA2 were not directly measured, and the findings were inferred from associations with serum CA2Ab levels. Therefore, future studies should perform a comprehensive evaluation of cellular immune and antibody responses. Fifth, the lack of pancreatic tissue analysis in KPD cases precluded direct confirmation of inflammatory changes in both the endocrine and exocrine regions. Regarding exocrine inflammation, pancreatic imaging or functional testing can help to address this limitation. Furthermore, as this study was conducted in a Japanese cohort, ethnic specificity must be considered, and replication in other populations is essential to establish generalizability.

In addition, the associations identified in this study—particularly those related to HLA-DRB1*08:03—were exploratory in nature and were not replicated in an independent cohort. Therefore, these findings should be regarded as hypothesis-generating rather than definitive. Moreover, although we observed an immunogenetic pattern that may be relevant to KPD, the present data do not establish whether this pattern is specific to this subtype or whether it contributes directly to its characteristic clinical presentation. To address these issues, future studies with larger, prospectively recruited cohorts and external validation will be essential.

In conclusion, HLA-DRB1*08:03 appears to play a pivotal role in KPD pathogenesis by bridging endocrine and exocrine immunological responses. Importantly, its presence may contribute less directly to β-cell impairment via B:9-23rPep-specific Th1 responses, potentially explaining the reversibility of β-cell dysfunction in the early stages of KPD. Future studies should elucidate the precise mechanisms connecting endocrine and exocrine immune responses in this disease.

## Data Availability

The datasets generated or analyzed in this study are available from the corresponding author upon request.

## Abbreviations

AT1D: acute-onset T1D
BMI: body mass index
CA2: carbonic anhydrase II
DK/DKA: diabetic ketosis/ketoacidosis
*HLA*: human leukocyte antigen
IFN: interferon
KPD: ketosis-prone type 2 diabetes
PBMCs: peripheral blood mononuclear cells
T1D: type 1 diabetes
Th1: T helper 1
SFCs: spot-forming cells
